# Mutagenesis of kiwifruit *CENTRORADIALIS*‐like genes transforms a climbing woody perennial with long juvenility and axillary flowering into a compact plant with rapid terminal flowering

**DOI:** 10.1111/pbi.13021

**Published:** 2018-10-25

**Authors:** Erika Varkonyi‐Gasic, Tianchi Wang, Charlotte Voogd, Subin Jeon, Revel S. M. Drummond, Andrew P. Gleave, Andrew C. Allan

**Affiliations:** ^1^ The New Zealand Institute for Plant & Food Research Limited (Plant & Food Research) Auckland New Zealand; ^2^ School of Biological Sciences University of Auckland Auckland New Zealand

**Keywords:** Actinidia, CEN, CENTRORADIALIS, CRISPR/Cas9, flowering, kiwifruit

## Abstract

Annualization of woody perennials has the potential to revolutionize the breeding and production of fruit crops and rapidly improve horticultural species. Kiwifruit (*Actinidia chinensis*) is a recently domesticated fruit crop with a short history of breeding and tremendous potential for improvement. Previously, multiple kiwifruit *CENTRORADIALIS* (*CEN*)‐like genes have been identified as potential repressors of flowering. In this study, CRISPR/Cas9‐ mediated manipulation enabled functional analysis of kiwifruit *CEN*‐like genes *AcCEN4* and *AcCEN*. Mutation of these genes transformed a climbing woody perennial, which develops axillary inflorescences after many years of juvenility, into a compact plant with rapid terminal flower and fruit development. The number of affected genes and alleles and severity of detected mutations correlated with the precocity and change in plant stature, suggesting that a bi‐allelic mutation of either *AcCEN4* or *AcCEN* may be sufficient for early flowering, whereas mutations affecting both genes further contributed to precocity and enhanced the compact growth habit. CRISPR/Cas9‐mediated mutagenesis of *AcCEN4* and *AcCEN* may be a valuable means to engineer *Actinidia* amenable for accelerated breeding, indoor farming and cultivation as an annual crop.

## Introduction

Flowering plants have adopted different life‐history strategies to survive adverse environmental conditions, and time their flowering for optimal reproduction. Annual and perennial growth habits are a consequence of different fates that can be acquired by meristems during the plant life cycle. Upon floral induction, vegetative shoot meristems undergo a transition to indeterminate inflorescence meristems, which give rise to determinate floral meristems. Unlike annuals, where all meristems are consumed within the same growing season, perennials maintain indeterminate meristems for the next season (Thomas *et al*., 2000). Fluctuations between annuality or perenniality have been observed (Bena *et al*., 1998; Hu *et al*., 2003; Tank and Olmstead, 2008) and selection for faster flowering and determinate growth habit underpins domestication of tender perennial crops such as tomato, which is commonly grown for production as an annual. However, many horticultural crop species are woody perennials, characterized by extended juvenile periods and large plant size, adapted to environments unlikely to favour natural mutations causing dramatic growth habit changes.

*Actinidia* are woody perennial species characterized by a relatively large genome with the basic chromosome number *x* = 29, ploidy variation, large plant size, climbing growth habit and excessive vigour. A genus comprising more than 50 species, *Actinidia* belong to the order Ericales, with all members being deciduous and dioecious with a temperate flowering phenology (Ferguson, 2016). A dichasium inflorescence or single flowers develop in the lower leaf axils of the shoots that emerge after winter dormancy and a juvenile unproductive period. The genus *Actinidia* includes several economically important horticultural species, commonly known as kiwifruit and widely recognized for health benefits. As a recently domesticated crop, few kiwifruit cultivars are the result of deliberate breeding programmes, being only one or two generations removed from the wild (Datson and Ferguson, 2011). Consequently, orchard management has many issues including functional dioecy, expensive management practices required to tame the long‐lived woody perennial climbers, inadequate crop yield in warmer climates, and susceptibility to diseases such as the devastating kiwifruit bacterial canker (Scortichini *et al*., 2012). Extended juvenility and long crossing cycles in particular are considerable constraints for genetic analysis and for creating improved cultivars. Newly established plants may take up to 5 years to grow a full canopy in the field (Ferguson, 2016), whereas *A. chinensis* cultivars commonly used for gene functional studies typically take 5 years to first flowering in glasshouse conditions.

Alteration of kiwifruit growth habit from a woody perennial climber into a short determinate plant with precocious flowering would be desirable to enable indoor growth, increase productivity and accelerate the breeding process. Genes involved in meristem transitions have been identified as good candidates for a switch in plant growth habit (Melzer, 2008), particularly PEBP (phosphatidylethanolamine‐binding protein) genes, homologs of *Arabidopsis FLOWERING LOCUS T* (*FT*) and *TERMINAL FLOWER1* (*TFL1*) (Wickland Daniel and Hanzawa, 2015 *)*. *FT*‐like genes are the key determinants of flowering time, and their antagonists *TFL1*‐like and *CENTRORADIALIS* (*CEN*)‐like are conserved repressors of flowering, acting in the shoot tip to maintain the indeterminate fate (Bradley *et al*., 1996, 1997; Lifschitz *et al*., 2014). Manipulations of FT and CEN availability and dosage provide means to manipulate developmental mechanisms; overexpression of *FT* genes (Klocko *et al*., 2016; Srinivasan *et al*., 2012; Wenzel *et al*., 2013) and down‐regulation of *TFL1/CEN* genes (Freiman *et al*., 2011; Kotoda *et al*., 2006; Yamagishi *et al*., 2016) have been advanced as tools for accelerated breeding. These approaches have often proven to be unreliable (Zhang *et al*., 2010) or failed to promote flowering in fruit crops, affecting other aspects of development instead (Freiman *et al*., 2015; Varkonyi‐Gasic *et al*., 2013). In contrast, stable mutations in these genes have underpinned domestication of crops such as legumes, sugar beet, sunflower and strawberry (Blackman *et al*., 2010; Iwata *et al*., 2012; Kwak *et al*., 2012; Liu *et al*., 2010; Pin *et al*., 2010), or in case of a natural mutation in the *CEN* homolog *SELF PRUNING* (Pnueli *et al*., 1998), revolutionized production and industrial processing of tomato.

Studies in diploid kiwifruit *A. chinensis* revealed three *FT* genes and five *CEN*‐like genes, with overexpression of *FT* genes resulting in extreme precocity, repression of vegetative growth and development of poorly differentiated *in vitro* flowers; in contrast, overexpression of all *Actinidia CEN*‐like genes delayed flowering in *Arabidopsis* and overexpression of *AcCEN* fully repressed flowering in *Actinidia eriantha* (Moss *et al*., 2018; Varkonyi‐Gasic *et al*., 2013; Voogd *et al*., 2017). We hypothesized that the multiple *CEN* homologs evolved to control the balance of vegetative growth and flowering in kiwifruit, to regulate the first onset of flowering, and terminal and axillary meristem identity. Genome editing using the CRISPR (clustered regularly interspaced short palindromic repeat)‐associated endonuclease Cas9 has proven a powerful and efficient tool for targeted mutagenesis, with the potential for editing multiple genomic loci and generating a range of mutations, affecting one or more alleles and gene family members (reviewed in (Komor *et al*., 2017). In this study, we utilized this approach to simultaneously target two kiwifruit *CEN*‐like genes *AcCEN4* and *AcCEN*. We demonstrate the role of these two genes in maturity, architecture and flowering time and generate plants amenable for rapid breeding cycles, urban farming and production as annuals. This study reveals the potential of fast‐tracked targeted improvement of a large, long‐lived woody perennial, with implications in the domestication and rapid improvement of non‐cultivated *Actinidia* and other long‐lived perennials, expanding horticultural crop diversity, and increasing sustainability of fruit production and food security.

## Results

### Identification of kiwifruit *CEN*‐like gene targets

**Figure 1:**
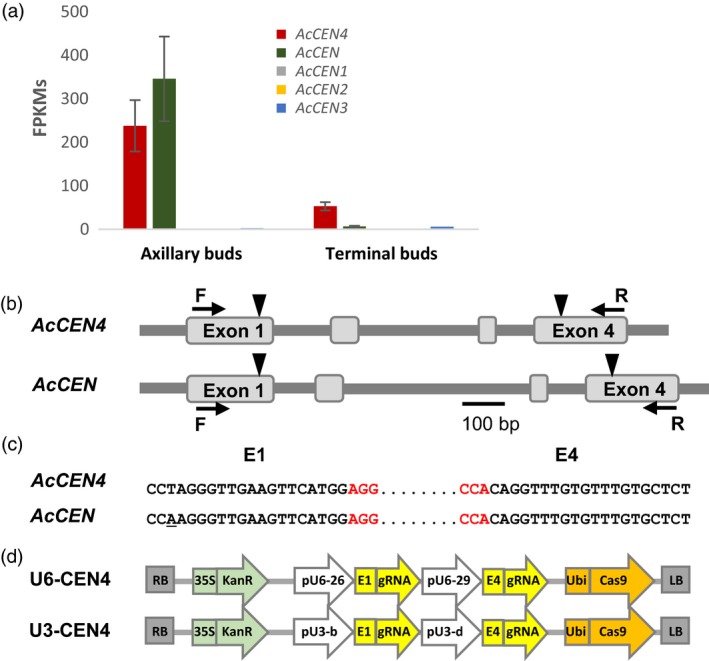
Selection of CRISPR/Cas9 target sequences and construct design. (a) Expression of *Actinidia chinensis CEN* genes in actively growing buds, presented as average fragments per kilobase of transcript per million reads (FPKM) ± SE of three (axillary buds) and two (terminal buds) biological replicates. (b) Schematic diagram of *A. chinensis CEN4* and *CEN* genes. Exons are presented as grey boxes, target sequence positions are indicated with arrowheads, gene‐specific forward (F) and reverse (R) oligonucleotide primer binding sites are indicated with arrows, PAM sequences are in red, and a single mismatch is underlined. (c) Alignment of the corresponding target E1 and E4 regions in *AcCEN4* and *AcCEN*. (d) Schematic diagram of the U6‐CEN4 and U3‐CEN4 constructs.

Kiwifruit has multiple *CEN*‐like genes which can act as conserved floral repressors (Voogd *et al*., 2017). Relatively high expression of *AcCEN4* and *AcCEN* in actively growing shoot tips and in latent axillary buds, respectively (Figure 1a), identified these two kiwifruit *CEN*‐like genes as prime candidates for regulation of kiwifruit architecture, maturity and flowering time. For the selection of CRISPR/Cas9 target sequences, alignment of *A. chinensis CEN* gene family cDNA sequences was used to identify homologous regions between *AcCEN4* and *AcCEN*. Two target sequences designated E1 and E4, in exon 1 and exon 4 of *AcCEN4*, respectively, were chosen based on full or almost full sequence identity between *AcCEN* and *AcCEN4*, but insufficient homology for targeting in the related PEBP genes (Figure S1). Two constructs which contained target sequences E1 and E4, each fused to generate target‐specific sgRNA sequences, were placed either under the control of *Arabidopsis* U6‐26 and U6‐29 promoters (U6‐CEN4), or *Arabidopsis* U3‐b and U3‐d promoters (U3‐CEN4), and introduced into a binary vector between the cauliflower mosaic virus (CAMV) *35S* promoter‐driven gene for kanamycin resistance and the *Cas9* gene driven by the parsley *Ubiquitin* promoter (Figure 1b–d, Figure S1). A construct containing the coding sequence of *Actinidia FT1* gene driven by the CaMV *35S* promoter (*35S:AcFT1*) was used to compare the effect of ectopic overexpression of a strong activator of flowering and mutagenesis of *CEN*‐like repressors of flowering.

### Differential effects of ectopic overexpression of *Actinidia FT1* and mutagenesis of *CEN*‐like genes on growth and flowering

**Figure 2:**
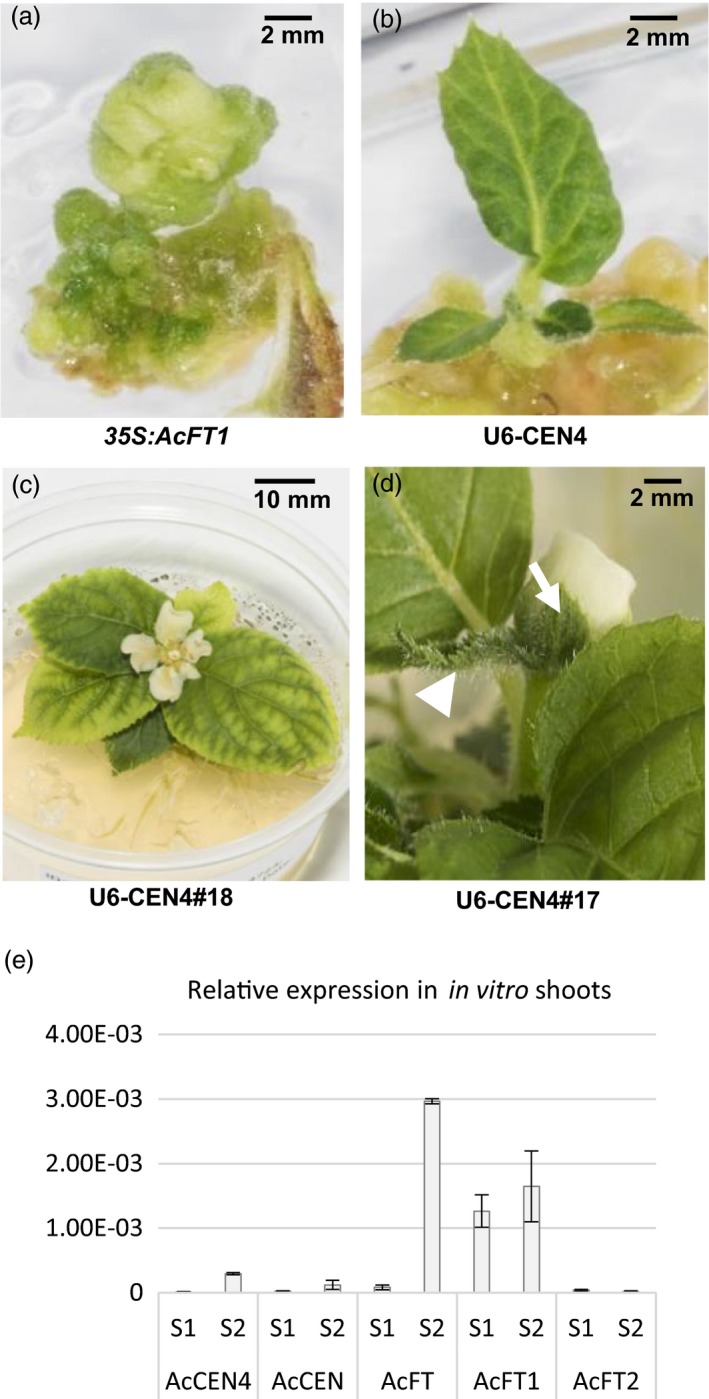
Precocity in *Actinidia chinensis* transgenic lines. (a) Ectopic overexpression of *Actinidia FT* gene (*35S:AcFT*1) gave rise to *in vitro* non‐viable flower development. (b) CRISPR/Cas9‐mediated mutagenesis of *AcCEN*4 and *AcCEN* (U6‐CEN4) gave rise to *in vitro* leaf development. (c) Early flowering in tissue culture in U6‐CEN4 lines. (d) The flower appearance is normal, with the exception of a subtending leaf‐like bract (arrowhead) and leaf‐like sepals (arrow). (e) Relative expression of *Actinidia CEN4*,*CEN* and *FT* genes during early *in vitro* growth after regeneration (S1) and in shoots of established stock plants (S2), normalized to kiwifruit *ACTIN* and presented as means ± SE of three biological replicates.

The U6‐CEN4, U3‐CEN4 and *35S:AcFT1* constructs were introduced into kiwifruit *A. chinensis* using standard regeneration and transformation protocols and kanamycin selection (Wang *et al*., 2007). This resulted in adventitious buds initiating 8 weeks post *Agrobacterium* co‐cultivation, giving rise to undifferentiated *in vitro* flowers with *35S:AcFT1* construct (Figure 2a). In contrast, all U6‐CEN4 and U3‐CEN4 lines demonstrated normal leaf development during *in vitro* growth (Figure 2b), giving rise to kanamycin‐resistant lines. PCR amplification and sequencing of *AcCEN4* gene fragments identified potential mutations in 75% (6/8) T0 U6‐CEN4 lines chosen for initial analysis (Figure S2) and a total of 25 lines were maintained for phenotypic evaluation. Possible mutations were also identified in 30% (3/10) T0 U3‐CEN4 lines. Three U6‐CEN4 lines (lines 14, 17 and 18) developed a single terminal flower 8–9 months after co‐cultivation with *Agrobacterium*, when plants had a minimum of six expanding leaves and established roots (Figure 2c). The flowers showed a mostly normal morphology, apart from a subtending leaf‐like bract and a leaf‐like appearance of one or two of the sepals (Figure 2d). These vegetative features of terminal flowers suggested an abrupt termination of vegetative growth, indicating a potential absence of an *FT* activator of flowering during the early stage of *in vitro* growth. To test this, expression analysis of tissue culture‐grown wild‐type *A. chinensis* was performed. Little or no expression of *AcFT* was detected in the early stage after regeneration, but the expression increased with shoot differentiation and appearance of the *AcCEN4* transcripts, whereas *AcFT1* was detected both in the early and later stage and *AcFT2* was absent (Figure 2e).

### Growth habit and reproductive development in precocious lines

**Figure 3:**
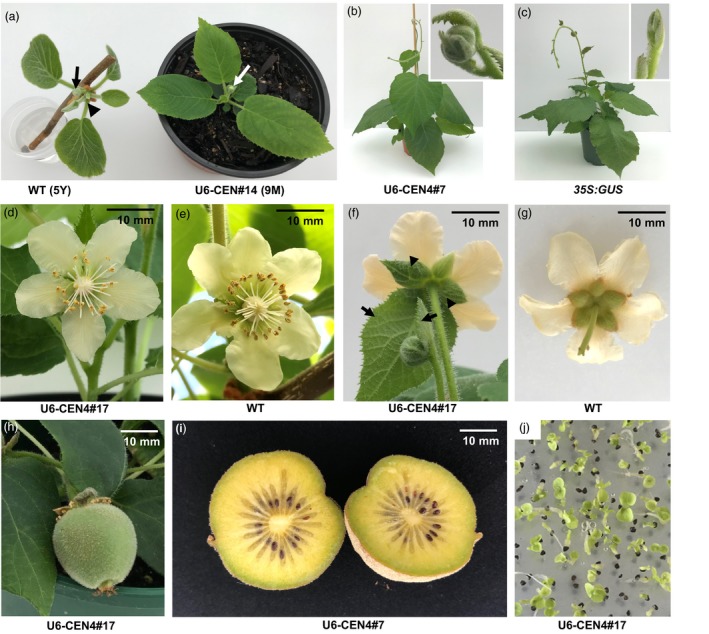
Accelerated flowering and normal reproductive development in *Actinidia chinensis* transgenic lines. (a) Terminal flower bud (white arrow) on U6‐CEN4 line 14 (right), compared to the vegetative terminal bud (black arrowhead) and axillary flower bud (black arrow) on an expanding shoot excised from a wild‐type (WT) *A. chinensis* plant (left). The age of the plant at first flowering is indicated; Y, year; M, month. (b, c) Appearance of U6‐CEN4 line 7 and a control *35S:GUS* transgenic *A. chinensis*. The inserts show close‐up images of a terminal flower bud and a vegetative shoot tip in line 7 and *35S:GUS* control respectively. (d, e) Normal appearance of the gynecium. (f, g) The subtending leaves (arrows) and leaf‐like sepals (arrowheads) on terminal flowers. (h) Developing fruit (30 days after pollination). (i) Mature fruit (120 days after pollination). (j) Seed germination.

When transplanted to soil, lines 14, 17 and 18 demonstrated a compact growth habit and terminal flowering after development of the minimum of six leaves (Figure 3a). Precocious flowering was further observed in U6‐CEN4 line 7, 12 months after co‐cultivation with *Agrobacterium*, with a terminal flower developing on a vine with 12 expanding leaves (Figure 3b,c). The morphology of the terminal flowers on all lines was similar, regardless of their precocity and growth habit. The number and appearance of floral organs was normal, with the exception of the subtending leaf and leaf‐like sepals (Figure 3d–g). The flowers were marginally smaller but developed a multi‐carpellate gynoecium comparable to wild‐type. Pollination using *A. chinensis* male pollen resulted in fruit set and development (Figure 3h,i), except in line 14, which failed to set fruit after multiple pollination attempts. Two fruit each from lines 17 and 18 and one from line 7 were harvested to evaluate whether they produced functional seed. All harvested fruit contained >100 seed, which were able to germinate (Figure 3j).

### Induced mutations in early flowering lines

**Figure 4:**
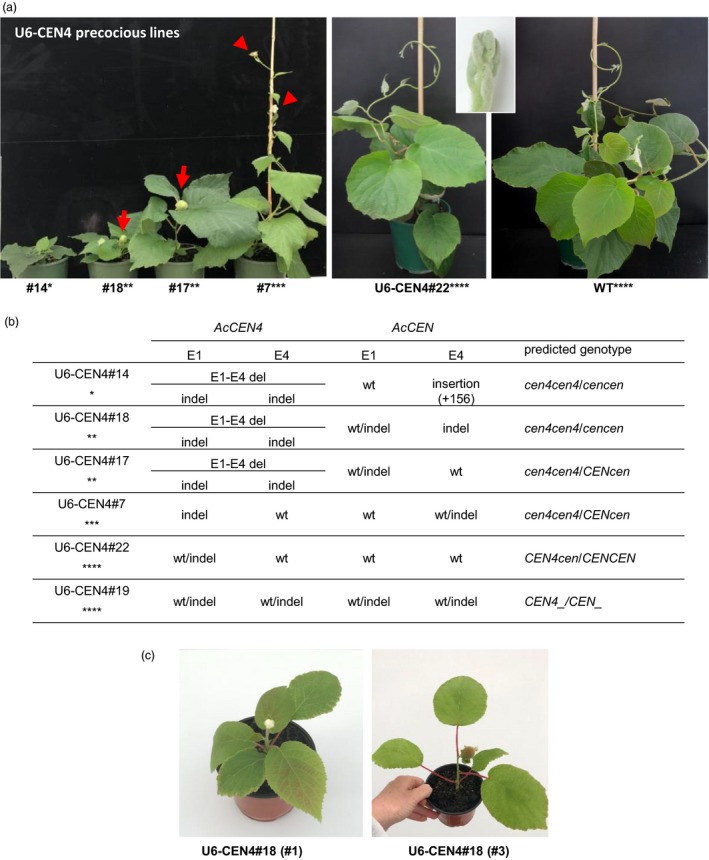
CRISPR/Cas9‐mediated editing of *AcCEN4* and *AcCEN* genes. (a) The height and architecture of U6‐CEN4 plants. Arrows and arrowheads indicate fruit and flowers respectively. The insert shows a close‐up image of the vegetative shoot tip in line 22. The asterisks denote the phenotypes: *, compact plant, early flowering, no fruit set; **, compact plant, early flowering; ***, vine habit, early flowering; ****, vine habit, no flowering. (b) Mutations identified in E1 and E4 sites in *AcCEN4* and *AcCEN* alleles. (c) Rapid flowering and fruit development in U6‐CEN4#18 lines after re‐establishment and propagation in tissue culture.

The initial PCR screen (Figure S2) identified mutations in *AcCEN4* gene in 75% and 30% of U6‐CEN4 and U3‐CEN4 T0 lines, respectively, but only four U6‐CEN4 T0 lines flowered early, three of which were extremely precocious and had a very compact growth habit (Figure 4a). To estimate the type and severity of mutations and the number of affected alleles, PCR amplification products were cloned and at least four clones were subjected to sequence analysis. Such genotyping identified mutations comprising small deletions or insertions in *AcCEN4* and *AcCEN* alleles in one or both of the E1 and E4 sites, at positions indicative of sgRNA‐directed Cas9 cleavage 3‐nt upstream of the protospacer adjacent motif (PAM) site (Figure 4b, Figures S2 and S3). A large deletion resulting from sgRNA‐directed Cas9 cleavage at both E1 and E4 regions in *AcCEN4* (E1‐E4 deletion) was detected in all compact early flowering lines (lines 14, 17 and 18), giving rise to a truncated gene in which the fragments of exon 1 and exon 4 were brought together without an introduction of a frame‐shift. In addition to this mutation, all compact lines had frame‐shifts in E1 and/or E4 regions of *AcCEN4*. Mutations were also identified in *AcCEN* alleles. In the most severely affected line 14, a large insertion of 156 nt in the last exon was identified in all *AcCEN* sequences, sharing homology to 26S rRNA. In line 18, frame shift mutations were identified in all *AcCEN* sequences at either both E1 and E4 sites or just E4 sites, but both wild‐type and mutant sequences were identified line 17, with a frame‐shift in *AcCEN* exon 1. In line 7, a frame shift in exon 1 was seen in all *AcCEN4* sequences and only in the last exon of some *AcCEN* sequences. A combination of mutant and wild‐type sequences in *AcCEN4* and *AcCEN* resulted in no visible alteration of growth and flowering time (Lines 19 and 22, Figure 4) and no mutations in related but very lowly expressed *AcCEN3* were detected in early flowering lines (Figure S2).

Different mutant sequences or a combination of wild‐type and mutant sequences identified in T0 lines suggested mutations occurring in single alleles, or genetic mosaics regenerated from cells of different genotypes in the same callus. Alternatively, additional mutagenesis was occurring through re‐editing of the target gene, because of the stable integration of *Cas9* gene and the sgRNA construct. The mosaicism or instability of mutations could affect phenotypes of plants multiplied by vegetative propagation, a common practice with woody perennials. To test if the appearance of plants remain stable with clonal propagation, one line (line 18) was re‐established in tissue culture, and eight new sublines were generated from adventitious buds regenerated from excised leaf tissue or axillary shoot tissue. A uniform compact size was seen in all clonally propagated lines and all lines flowered and were capable of bearing fruit after developing 4‐6 leaves, demonstrating that vegetative multiplication gives rise to comparable precocious plants (Figure 4c).

### Bi‐allelic mutation of either *AcCEN4* or *AcCEN* may be sufficient for early flowering

**Figure 5:**
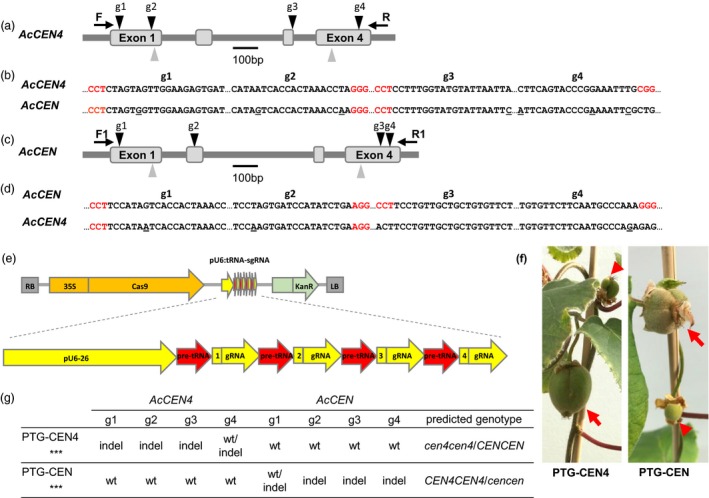
Mutagenesis of *AcCEN4* and *AcCEN* using polycistronic tRNA‐sgRNA (PTG) constructs. (a‐e) Selection of CRISPR/Cas9 target sequences and polycistronic tRNA‐sgRNA (PTG) construct design: schematic diagram of *AcCEN4* gene with target sequences *AcCEN4* g1‐4 (a), alignment of *AcCEN4* g1‐4 target sequences and corresponding *AcCEN* regions (b), schematic diagram of *AcCEN* gene with target sequences *AcCEN* g1‐4 (c), alignment of *AcCEN* g1‐4 target sequences and corresponding *AcCEN4* regions, and a schematic diagram of the PTG construct (e). Exons are presented as grey boxes, target sequence positions are indicated with black arrowheads, gene‐specific forward (F) and reverse (R) oligonucleotide primer binding sites are indicated with arrows, PAM sequences are in red, and mismatches are underlined. Grey arrowheads indicate E1 and E4 sequences targeted by U6‐CEN4 constructs. (f) Fruit developing in terminal and axillary positions on precocious PTG lines. (g) Gene‐specific mutations identified in the PTG‐CEN4 and PTG‐CEN lines.

The genotyping of lines 17 and 7 suggested that bi‐allelic mutagenesis of *AcCEN4* may be sufficient for early flowering. In line 7, a combination of wild‐type sequences and a frame shift affecting only the C‐terminal region of predicted AcCEN were detected (Figure 4b, Figure S3). Mutations in *AcCEN* potentially contributed to precocity and short plant stature seen in compact lines 14, 17 and 18, indicating that mutation of *AcCEN* may also result in early flowering. To address this hypothesis, additional gene‐specific target sequences were identified (Figure 5a–d). Two polycistronic tRNA‐sgRNA (PTG) cassettes, each with four sgRNA sequences preferentially targeting *AcCEN4* (PTG‐CEN4) or *AcCEN* (PTG‐CEN), were placed under the control of *Arabidopsis* U6‐26 promoter, introduced into a binary vector between the *35S* promoter‐driven *Cas9* gene and the gene for kanamycin resistance (Figure 5e) and transformed into kiwifruit *A. chinensis*. More than 30 lines were generated for each construct and grown in tissue culture, and 20 lines per construct were transplanted to soil and maintained for phenotypic evaluation. Precocious flowering was observed in a PTG‐CEN4 and a PTG‐CEN line on vines with 15 leaves, giving rise to fruit after pollination (Figure 5f). Genotyping of these lines identified small deletions or insertions in target sites, resulting in frame‐shifts in *AcCEN4* alleles in the precocious PTG‐CEN4 line and frame‐shifts in *AcCEN* alleles in the precocious PTG‐CEN line (Figure 5g, Figure S3). Therefore, mutagenesis of both alleles of either of the genes resulted in a determinate phenotype with early flowering, yet not as early as seen with mutations in both genes.

### Altered architecture, continuous flowering and dormancy

**Figure 6:**
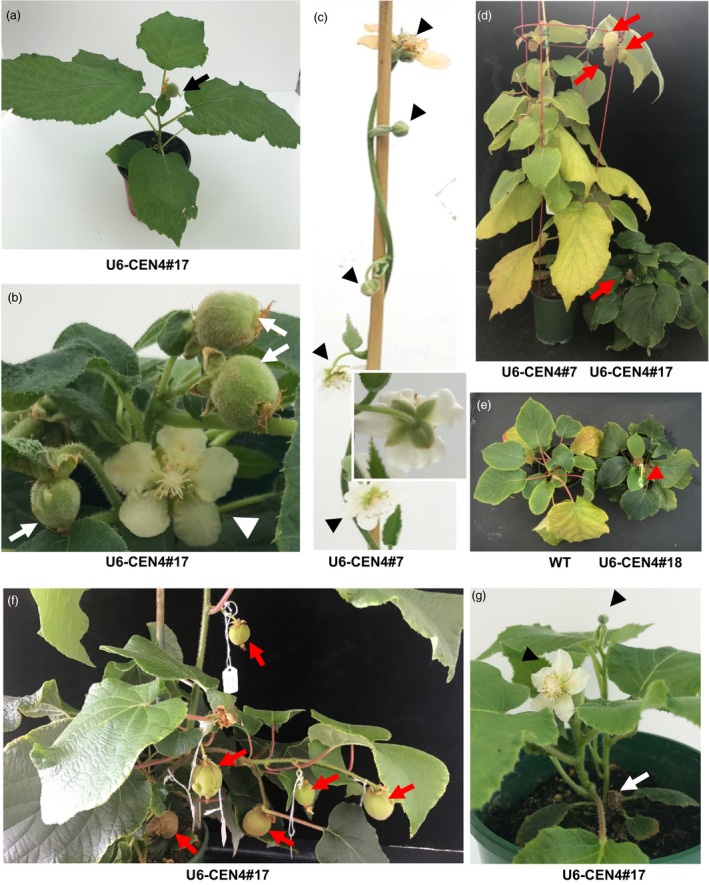
Pleiotropic phenotypes of precocious kiwifruit. (a) Single terminal fruit on a compact plant (arrow). (b) Simultaneous flower (arrowhead) and fruit (arrows) development on a compact plant. (c) Vine growth habit with terminal (arrow) and axillary (arrowheads) flower development on line 7. The insert shows the normal sepals on an axillary flower. (d, e) Differential responses to dormancy‐inducing conditions. Developing fruit and the short shoot with terminal flower bud are indicated by arrows and an arrowhead respectively. (f) Internode elongation and multiple fruit developing in terminal and axillary positions on lateral shoots emerging from the base of the trunk. (g) Basal shoots with terminal flowers (arrowheads) emerging after hard pruning (arrow denotes where the shoot was removed).

In addition to premature flowering, the lines exhibited pleiotropic phenotypes including altered architecture and dormancy requirement. The developing terminal flower or fruit on compact early flowering lines was sufficient to prevent axillary bud outgrowth (Figure 6a), but their senescence or removal resulted in outgrowth of axillary shoots with terminal flowers, enabling simultaneous flower and fruit development on the stunted, bushy plant (Figure 6b). The U6‐CEN4 line 7 and the precocious PTG lines were capable of developing axillary flowers in addition to the terminal flower (Figures 5f and 6c), giving rise to multiple fruit on a single shoot (Figure 6d). In these lines, shortening of day length induced bud set and leaf senescence comparable to control plants, whereas the compact plants remained insensitive to changes in day length and did not enter short‐day induced dormancy. Instead, compact lines 14, 17 and 18 remained green and continued to develop new shoots with terminal flowers (Figure 6d, e). Later development in initially compact and vine lines was similar, with repeated cycles of lateral branch extension and flowering. The newly emerging lateral branches arising from vegetative buds at the base of the trunk bore longer internodes and commonly developed axillary flowers, producing multiple fruit after pollination (Figure 6f). Hard pruning resulted in emergence of new short shoots with terminal flowers (Figure 6g), followed by repeated cycles of lateral branch growth and flowering, ensuring continual production.

### Analysis of the heterozygous T1 lines

**Figure 7:**
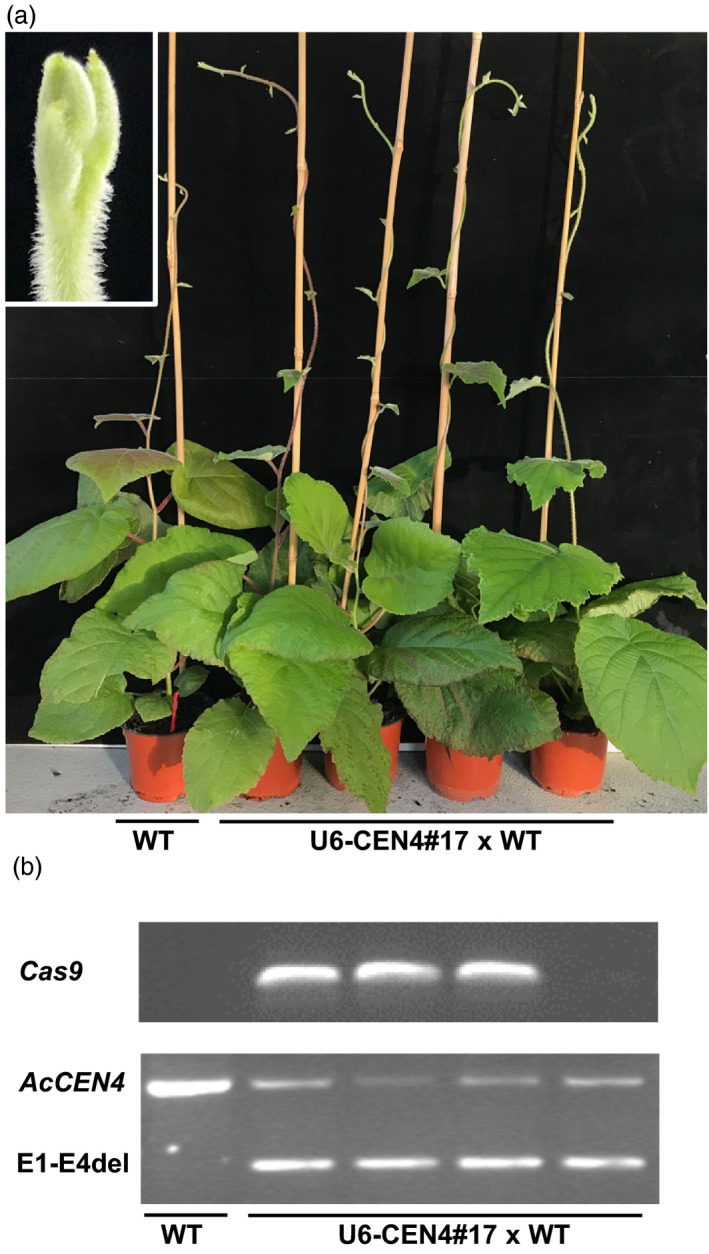
Heterozygous progeny displays long juvenility. (a) The appearance of plants. The insert shows a close‐up image of the vegetative shoot tip of a representative plant. (b) Genotyping identifies lines carrying *Cas9* and *AcCEN4* E1‐E4 deletion.

Because of the dioecious nature of *Actinidia*, pollination was performed using pollen from a wild‐type male diploid *A. chinensis*, carrying the wild‐type alleles of *AcCEN4* and *AcCEN* and giving rise to heterozygous progeny. To determine whether the early flowering trait could be efficiently transferred to the next generation through editing in the Cas9‐sgRNA background after fertilization, seeds from a fruit of line 17 were germinated. A total of 80 germinated seedlings were scored for flowering for 4 months following germination, at which stage they produced between six and 15 leaves, reaching the flowering stage of compact and vine‐like lines respectively. None of the lines flowered at this stage (Figure 7a), despite of the presence of *Cas9* and the *AcCEN4* E1‐E4 deletion in the genome (Figure 7b). Therefore, the progeny in an outcrossing perennial kiwifruit remains largely heterozygous and flowers late when U6‐CEN4 construct is used.

## Discussion

### *AcCEN4* and *AcCEN* regulate the life cycle, height and architecture of kiwifruit

Previously, five kiwifruit *CEN*‐like genes have been identified (Voogd *et al*., 2017). This gene duplication and divergence would be expected to contribute to the regulation of kiwifruit architecture and flowering, but it has been highly problematic to differentiate their endogenous roles, functionally distinguish paralogous genes and generate mutations specifically affecting one or more alleles and members of this gene family. Genome editing can now circumvent some of these problems. Here, we combined the potential for editing single and multiple genomic loci by CRISPR/Cas9 and the ability to transform the diploid *A. chinensis*, to address the hypothesis that *AcCEN4* and *AcCEN* regulate the flowering time, life cycle and architecture of kiwifruit.

The number of affected genes and alleles generally correlated with the precocity and change in plant stature. Mutagenesis of both alleles of either *AcCEN4* or *AcCEN* gave rise to a determinate phenotype with early flowering, whereas mutations in both genes resulted in even earlier flowering, resembling the highly compact habit and very early yields in double‐determinate tomato, a consequence of mutations in two PEBP genes with flower‐repressing activities (Fridman *et al*., 2002; Soyk *et al*., 2016). The type of mutations and position of frame shifts were likely to impact on the phenotypes. Small changes in *FT* and *TFL1/CEN*‐like sequences can reverse their activating or repressing roles in regulation of flowering (Ahn *et al*., 2006; Ho and Weigel, 2014) and can interfere with flower‐activating capacity of other members of the PEBP family (Blackman *et al*., 2010; Pin *et al*., 2010). All compact lines had the *AcCEN4* E1‐E4 deletion and a large insertion was seen in *AcCEN* that may have rendered line 14 infertile, although it produced terminal flowers indistinguishable from the fertile terminal flowers developing on other early flowering lines. Architecture ranging from compact plants to vines is most likely the outcome of the rate of termination. The very early flowering lines were stunted, although more vigorous growth and internode expansion was seen in lateral shoots emerging later, giving rise to bushy plants with terminal and axillary flowers. The altered response to dormancy‐inducing conditions was prominent in very early flowering lines, which remained green and continued to produce new shoots, with a brief pause in growth during fruit development. These phenotypes, particularly the altered architecture with increased lateral branching and insensitivity to environmental conditions initiating bud‐set and dormancy, were in common with trees overexpressing *FT* genes (Endo *et al*., 2005; Klocko *et al*., 2016; Srinivasan *et al*., 2012), demonstrating common outcomes of altered FT/CEN balances. While ectopic overexpression of kiwifruit *FT* genes gave rise to *in vitro* flowers (Moss *et al*., 2018; Voogd *et al*., 2017), the terminating effect of mutations in *AcCEN4* and *AcCEN* became evident in the stage when the flowering activator *AcFT* expression was detectable, suggesting that the mutations affecting *CEN* genes are only relevant in the presence of a functional FT, as previously emphasized in tomato (Lifschitz *et al*., 2014).

The shoot character of terminal flowers was indicative of an abrupt abortion of vegetative growth but insufficient activation of floral fate. It is possible that mutations in *CEN*‐like genes may not fully activate floral meristem identity genes, particularly if an FT activator of flowering is not sufficiently expressed or perhaps not translocated before the vascular connections and sink‐source relationship are firmly established. In contrast, the axillary flowers were bractless (no subtending leaf) and with normal sepals, suggesting a fully acquired floral fate, despite their unusual position in the upper leaf axils. In wild‐type *A. chinensis* these positions are occupied by latent buds, which have the capacity to develop into inflorescence‐bearing shoots in the following spring, after sufficient winter chilling is perceived during winter dormancy (Walton *et al*., 1997). Development of single flowers in these positions suggests that mutations in *CEN*‐like genes drive determinacy and offset the need for dormancy and winter chilling in axillary meristems, and the fully acquired floral fate in these flowers imply that additional mechanisms may contribute to activate floral meristem identity genes.

### Engineering determinacy, altered architecture and rapid flowering for accelerated breeding and urban farming

The ability to convert perennial crops into annual production and grow plants in confined spaces is becoming increasingly important with predicted effects of climate change and population growth. Indoor farming is being adopted for annual production of leafy vegetables and tender perennial crops such as tomato, selected over thousands of years of domestication and breeding for the compact determinate growth habit and day‐neutral flowering (Soyk *et al*., 2016). However, large plant size and long juvenility of woody perennial horticultural species have been an obstacle for indoor cultivation of most fruit crops.

Here we engineered determinacy, compact size and rapid flowering in kiwifruit, using CRISPR/Cas9‐mediated mutagenesis of *CEN*‐like candidate genes, enabling a woody perennial to complete its life cycle within a year. Genome editing with the U6‐CEN4 construct was found to be relatively efficient. A high mutation rate was detected and four of the 25 U6‐CEN4 lines flowered early, with mutations detected in one or both *AcCEN4* and *AcCEN* and in one or both of the E1 and E4 target sites. Analysis of T0 U3‐CEN4 lines suggested a lower editing efficiency in kiwifruit when sgRNA sequences were driven by *Arabidopsis* U3 promoters. Similarly, the excess of 40 lines carrying the PTG constructs gave rise to only two lines with bi‐allelic mutations and rapid flowering phenotypes. This may be at least partly caused by the selected CRISPR/Cas9 target sequences chosen to preferentially target single genes, rather than the PTG construct design, already shown as successful in kiwifruit (Wang *et al*., 2018).

The late flowering in the heterozygous progeny suggests no or very low frequency of heritable gene targeting in kiwifruit, at least when using the U6‐CEN4 editing construct, in which the *Arabidopsis* U6 and the parsley *Ubiquitin* promoters drive the expression of sgRNAs and *Cas9* respectively. A different set of promoters better suited for specific expression in *Actinidia* may facilitate genome editing after pollination and increase the heritability of the early flowering trait. In *Arabidopsis*, the expression of *Cas9* from germline‐specific promoters increased the efficiency of CRISPR/Cas9‐mediated gene editing and the frequency of heritable gene targeting (Miki *et al*., 2018; Yanfei *et al*., 2016), hence a similar approach could be used for kiwifruit. Alternatively, generation of early flowering male and female parents using CRISPR/Cas9 mutagenesis of *CEN* genes could overcome the prolonged T1 generation time in perennials that require cross‐pollination to produce fruit, typical of kiwifruit and many other horticultural crops.

Kiwifruit domestication started in the early 20th century. Increasingly popular, well known for high nutritional value and as a rich source of healthy compounds (Ampomah‐Dwamena *et al*., 2009; Bulley and Laing, 2016; Montefiori *et al*., 2005; Park *et al*., 2013), kiwifruit has a tremendous potential for further improvement. The approach developed in this study shows a promising pathway to quickly generate new varieties, either by accelerated breeding with rapid flowering parents or targeted gene editing in already available selections. The plants generated in this study can be used as model plants in furthering detailed understanding of mechanisms and processes in kiwifruit, including fruit development and plant‐pathogen studies. In addition, CRISPR/Cas9‐mediated mutagenesis of *AcCEN4* and *AcCEN* homologs paves the way to domesticate and improve non‐cultivated *Actinidia*.

In conclusion, editing of *Actinidia CEN4* and *CEN* genes converts kiwifruit into a compact plant with determinate growth habit and terminal flowering, amenable to growth in confined spaces, and providing the opportunity to cultivate kiwifruit as an annual crop. The fast life cycle holds the potential to dramatically accelerate targeted breeding, whereas optimization of growing conditions for faster growth and indoor farming could increase productivity and enable cropping in any geographical and climatic conditions. This approach could be a useful tool to accelerate the development of new varieties across the horticultural sector, whereas the precise adjustment of plant architecture offers possibilities for changes in the way in which new varieties are bred and cultivated.

## Materials and methods

### Plant material

Plant material from a female kiwifruit cultivar ‘Hort16A’ (*A. chinensis* Planch. var. *chinensis*) was used for transformation and expression studies. The media were described before (Wang *et al*., 2007). Pollination of flowers was performed using pollen collected from male diploid *A. chinensis*. The male plants were container‐raised in a containment greenhouse at Plant & Food Research, Auckland, New Zealand. The pollen was collected in spring 2016 and stored in the freezer for 1 year.

### Expression analysis

The RNAseq experiment was described before (Voogd *et al*., 2017). Briefly, *A. chinensis* axillary and terminal buds were collected at regular intervals over the season, subjected to RNA‐seq, and uniquely mapped reads with no mismatches per paired alignment were used to determine gene expression. The data were interrogated for expression of *Actinidia CEN*‐like genes in axillary and terminal buds during active growth and presented as average fragments per kilobase of transcript per million reads (FPKM) ± SE of three (axillary buds) and two (terminal buds) biological replicates.

For RT‐qPCR studies, total RNA was isolated using the Spectrum Plant Total RNA Kit (Sigma‐Aldrich). An aliquot of 1 μg RNA was reverse transcribed using the QuantiTect Reverse Transcription Kit (Qiagen) and quantification using real‐time PCR were performed with the FastStart DNA Master SYBR Green I mix (Roche Diagnostics) using the LightCycler 1.5 instrument and the LightCycler Software version 4 (Roche Diagnostics). Amplification was carried out using a 10^−3^ dilution of the cDNA template, with an initial denaturing step at 95 °C for 5 min, then 40 cycles of 95 °C for 5 s, 60 °C for 5 s, and 72 °C for 10 s. A non‐template control was included in each run. Oligonucleotide primers (Table S1) were designed to produce amplification products of 100–150 nucleotides. The specificity of primer pairs was confirmed by melting curve analysis of PCR products and agarose gel electrophoresis followed by sequence analysis. The expression was normalized to kiwifruit *ACTIN* (GenBank accession FG403300) and presented as a mean ± SE of three biological replicates.

### Target identification and vector construction

*Actinidia chinensis* PEBP gene family coding region sequences were aligned using Geneious ClustalW Alignment (Geneious, Biomatters Ltd, version 8.1.2) (www.geneious.com). Two target sequences designated E1 and E4, in exon 1 and exon 4 of *AcCEN4*, respectively, were chosen based on full or almost full sequence identity between *AcCEN* and *AcCEN4*, but insufficient homology for targeting in the related PEBP genes (Figure S1). BlastN alignment using the *A. chinensis* genome (Huang *et al*., 2013; Pilkington *et al*., 2018) was used to confirm absence of other highly homologous sequences, minimizing the potential for off‐target editing. Two constructs were designed which contained target sequences E1 and E4, each fused to generate target‐specific sgRNA sequences, and placed either under the control of *Arabidopsis* U6‐26 and U6‐29 promoters, or *Arabidopsis* U3‐b and U3‐d promoters. When required, additional nucleotides were added to ensure correct initiation of target sequence transcription from U6 and U3 promoters (Figure S1). Gateway recombination sites were added on each end and the resulting 1150 nt and 868 nt DNA fragments designated U6‐CEN4 and U3‐CEN4, respectively, were synthesized (Genewiz, South Plainfield, NJ; www.genewiz.com). The synthetic DNA was recombined between the CaMV *35S* promoter‐driven gene for kanamycin resistance and the *Cas9* gene driven by the parsley *Ubiquitin* promoter, in vector pDE‐Cas9(KanR) modified from pDE‐Cas9 by replacing the *Hin*dIII fragment carrying the plant selection cassette with that from pDE‐Cas9(D10A) (Fauser *et al*., 2014). Specific targeting of *AcCEN4* and *AcCEN* genes was achieved using the tandemly arrayed tRNA‐gRNA structure described by Xie *et al*. (2015), using an optimized sgRNA design (Dang *et al*., 2015) and target selection criteria based on Doench *et al*. (2014). The constructs containing the polycistronic tRNA‐sgRNA cassette placed downstream of the *Arabidopsis thaliana* U6‐26 promoter, with Gateway recombination sites added on each end, were synthesized (Genewiz) and recombined with the destination vector pDE‐KRS, derived from pHEX2, which expresses Cas9 from the *35S* promoter and contains the plant kanamacyn resistance gene. The *35S:AcFT1* construct and the *35S:GUS* vector control were described previously (Voogd *et al*., 2017). All resulting plasmids were transformed into *Agrobacterium tumefaciens* strain EHA105 by electroporation.

### Plant transformation and growth

*Agrobacterium tumefaciens* mediated transformation of *A. chinensis* was performed as previously described (Wang *et al*., 2007). Briefly, leaf strips excised from *in vitro*‐grown shoots were co‐cultivated with *Agrobacterium* suspension culture and transferred to regeneration and selection medium. Individual calli were excised from the leaf strips for further selection and bud induction, and adventitious buds regenerated from the calli were excised and transferred to shoot elongation medium. When shoots had grown to 1–2 cm high, they were transplanted onto rooting medium. Rooted transgenic plants were then potted and grown in a containment greenhouse at ambient conditions (temperature min 18 °C/ max 30 °C night/day, 14 h/10 h light/dark in summer, with gradual day length shortening to 12 h/12 h light/dark at the beginning of autumn). For clonal propagation, leaf tissue was surface‐sterilized and allowed to regenerate and develop roots as described, then grown in a containment greenhouse as described.

### Seed germination

Seed was collected from immature fruit 60 days after pollination or mature fruit 120 days after pollination. The fruit was surface‐sterilized by soaking in 1.5% (v/v) sodium hypochlorite and 0.1% (v/v) Tween 20 for 15 min before seed extraction, after which the seed was further sterilized for 10 min. Seeds were germinated on a medium supplemented with coconut milk and casein hydrolysate (Table S2), before transplanting into soil for growth in the glasshouse (18 °C night, 28 °C day) at 18 h light/ 6 h dark cycles.

### Genotyping

Genomic DNA was extracted from kanamycin resistant kiwifruit explants and glasshouse‐grown plants using the DNeasy Plant Mini Kit (Qiagen) following the manufacturer's instructions. For fast evaluation of lines for editing events, PCR amplification was performed using the iProof High‐Fidelity DNA Polymerase (Bio‐Rad) using gene‐specific oligonucleotide primers (Table S1), with an initial denaturing step at 95 °C for 5 min, then 35 cycles of 95 °C for 15 s, 58 °C for 15 s, and 72 °C for 1 min. Amplification products were purified using the DNA Clean & Concentrator‐5 kit (Zymo Research) and sequenced using gene‐specific oligonucleotide primers. For subsequent verification, amplification products were cloned into pGEM‐T Easy (Promega) and at least four clones of each line were subjected to sequence analysis. Oligonucleotide primer synthesis and sequencing were performed by Macrogen (www.macrogen.com).

## Declarations

The authors declare no competing financial interests.

## Contributions

EV‐G and ACA conceived the study and led the research. EV‐G designed the constructs, generated transgenic plants and wrote the manuscript. TW generated, maintained and pollinated the transgenic plants. CV designed the constructs and performed the cloning. SJ and EV‐G performed the genotyping and RT‐qPCR studies. RSMD and APG designed the vectors and contributed to the experimental design. All authors discussed the results and reviewed and contributed to the manuscript.

## Supporting information


**Figure S1** Selection of CRISPR/Cas9 target sequences and construct design. (a) Alignment of the corresponding target E1 and E4 regions in *AcCEN4*,* AcCEN* and other *Actinidia chinensis CEN*,* BROTHER OF FT* (*BFT*), *MOTHER OF FT* (*MFT*) and *FT* genes. PAM sequence is in red and mismatches are highlighted in yellow. (b) The sequence of constructs used for CRISPR/Cas9‐mediated editing of *AcCEN4* and *AcCEN* genes. Green, gene‐specific target sequence (protospacer); red, guide RNA (crRNA, linker loop and tracrRNA); blue, end of the promoter sequence; underlined, +1 position (appropriate nucleotide added where necessary, G and A for pU6 and pU3 respectively).
**Figure S2** Evaluation of CRISPR/Cas9‐mediated gene editing in transgenic kiwifruit. (a) Example of amplification using gene‐specific primers, which identified the E1‐E4 deletion in *AcCEN4* in lines 17 and 18 (arrow). (b) Examples of wild‐type (top) and edited *AcCEN4* E1 regions identified by sequencing of the PCR fragment. (c) Wild‐type *AcCEN3* E1 and E4 regions identified by sequencing of the PCR fragment in early flowering line 17.
**Figure S3** Mutations (red font) identified in *AcCEN4* and *AcCEN* alleles in U6‐CEN4 (a), PTG‐CEN4 (b) and PTG‐CEN (c) lines. At least four clones of gene‐specific amplification products were subjected to sequence analysis. wt, wild‐type; +, insertion; ‐, deletion.

**Table S1** Oligonucleotide primer sequences used in this study.

**Table S2** Medium for seed germination.
