# Supplementary material for: Mutagenesis of kiwifruit CENTRORADIALIS ‐like genes transforms a climbing woody perennial with long juvenility and axillary flowering into a compact plant with rapid terminal flowering
Source: Plant Biotechnol J. 2018 Oct 25;17(5):869–80. doi: 10.1111/pbi.13021 (PMC6587708; doi:10.1111/pbi.13021)
Supplement: Supplementary file 1 — Figure S1 Selection of CRISPR/Cas9 target sequences and construct design. (a) Alignment of the corresponding target E1 and E4 regions in AcCEN4, AcCEN and other Actinidia chinensis CEN, BROTHER OF FT (BFT), MOTHER OF FT (MFT) and FT genes. PAM sequence is in red and mismatches are highlighted in yellow. (b) The sequence of constructs used for CRISPR/Cas9‐mediated editing of AcCEN4 and AcCEN genes. Green, gene‐specific target sequence (protospacer); red, guide RNA (crRNA, linker loop and tracrRNA); blue, end of the promoter sequence; underlined, +1 position (appropriate nucleotide added where necessary, G and A for pU6 and pU3 respectively). Figure S2 Evaluation of CRISPR/Cas9‐mediated gene editing in transgenic kiwifruit. (a) Example of amplification using gene‐specific primers, which identified the E1‐E4 deletion in AcCEN4 in lines 17 and 18 (arrow). (b) Examples of wild‐type (top) and edited AcCEN4 E1 regions identified by sequencing of the PCR fragment. (c) Wild‐type AcCEN3 E1 and E4 regions identified by sequencing of the PCR fragment in early flowering line 17. Figure S3 Mutations (red font) identified in AcCEN4 and AcCEN alleles in U6‐CEN4 (a), PTG‐CEN4 (b) and PTG‐CEN (c) lines. At least four clones of gene‐specific amplification products were subjected to sequence analysis. wt, wild‐type; +, insertion; ‐, deletion. [file PBI-17-869-s002.pdf]

Figure S1

(a)

	E1		E4
AcCEN4	CCTAGGGTTGAAGTTCATGGAGG	....	CCACAGGTTTGTGTTTGTGCTCT
AcCEN	CCAAGGGTTGAAGTTCATGGAGG	....	CCACAGGTTTGTGTTTGTGCTCT
AcCEN1	CCTAGGGTAGAAATCCAAGGAGG	....	CCACAGGTTTGTGTTTGTTCTCT
AcCEN2	CCTAGAGTTGAAGTCCATGGAGG	....	CCACAGGTTTGTGTTTGTTCTGT
AcCEN3	CCTAGAGTTGAAGTCCATGGAGG	....	CCACAGGTTTGTGTTTGTTCTGT
AcBFT1	GCTCGCGTAGAGATCGGTGGAGA	....	CCACCGGTACGTGTTCATCTTGT
AcBFT2	CCTCGCGTAGAGATCGGCGGTGA	....	CCACCGATACGTGTTCTTCTTGT
AcBFT3	CCTCGCGTAGAGATCGGTGGTGA	....	CCACCGGTACGTGTTCCTCTTGT
AcFT	CCGAGGGTTGATATTGGTGGCGA	....	CCATCGCTTTGTTCTGGTGCTAT
AcFT1	CCTAGAGTTGATGTTGGAGGTGA	....	TCATCGCTTTGTGTTCGTGTTGT
AcFT2	CCTAGGGTTGATATTGGAGGTGA	....	TCATCGCTTTGTGTTCGTGTTGT
AcMFT1	CCTAGGGTCACCATCTCTGGTCA	....	CCACAGGTATATACTGGTCCTCT
AcMFT2	CCTAGGGTCACAATCACTGGCCA	....	ACATCGCTGCATCCTGCTTCTGT

(b) U6-CEN4 construct (1150 nt):

attB1-GCT-pU6-26 (387 bp)-

GATTGAGAGCACAAACACAAACCTGGTTTTAGAGCTAGAAATAGCAAGTTAAAATAAGGCTAGTCCGTTATCA

ACTTGAAAAAGTGGCACCGAGTCGGTGCTTTTTTTCAAGAGCTTGGAGTGGATGGAATCGGCAGCAAAGGA-

pU6-29 (319 bp)-

GATTGCCTAGGGTTGAAGTTCATGGGTTTTAGAGCTAGAAATAGCAAGTTAAAATAAGGCTAGTCCGTTATCA

ACTTGAAAAAGTGGCACCGAGTCGGTGCTTTTTTTACCc-attL2

U3-CEN4 construct (868 nt):

attB1-GCT-pU3-b(324 bp)-

GGTCAGAGCACAAACACAAACCTGGTTTTAGAGCTAGAAATAGCAAGTTAAAATAAGGCTAGTCCGTTATCAA

CTTGAAAAAGTGGCACCGAGTCGGTGCTTTTTTTCAAGAGCTTGGAGTGGATGGAATCGGCAGCAAAGGA-

pU3-d (101 bp)-

GGTCACCTAGGGTTGAAGTTCATGGGTTTTAGAGCTAGAAATAGCAAGTTAAAATAAGGCTAGTCCGTTATCA

ACTTGAAAAAGTGGCACCGAGTCGGTGCTTTTTTTACC-attL2

Supplementary Figure S1. Selection of CRISPR/Cas9 target sequences and construct design. (a) Alignment of the corresponding target E1 and E4 regions in *AcCEN4*, *AcCEN* and other *A. chinensis CEN*, *BROTHER OF FT* (*BFT*), *MOTHER OF FT* (*MFT*) and *FT* genes. PAM sequence is in red and mismatches are highlighted in yellow. (b) The sequence of constructs used for CRISPR/Cas9-mediated editing of *AcCEN4* and *AcCEN* genes. Green, gene-specific target sequence (protospacer); red, guide RNA (crRNA, linker loop and tracrRNA); blue, end of the promoter sequence; underlined, +1 position (appropriate nucleotide added where necessary, G and A for pU6 and pU3, respectively).

Figure S2

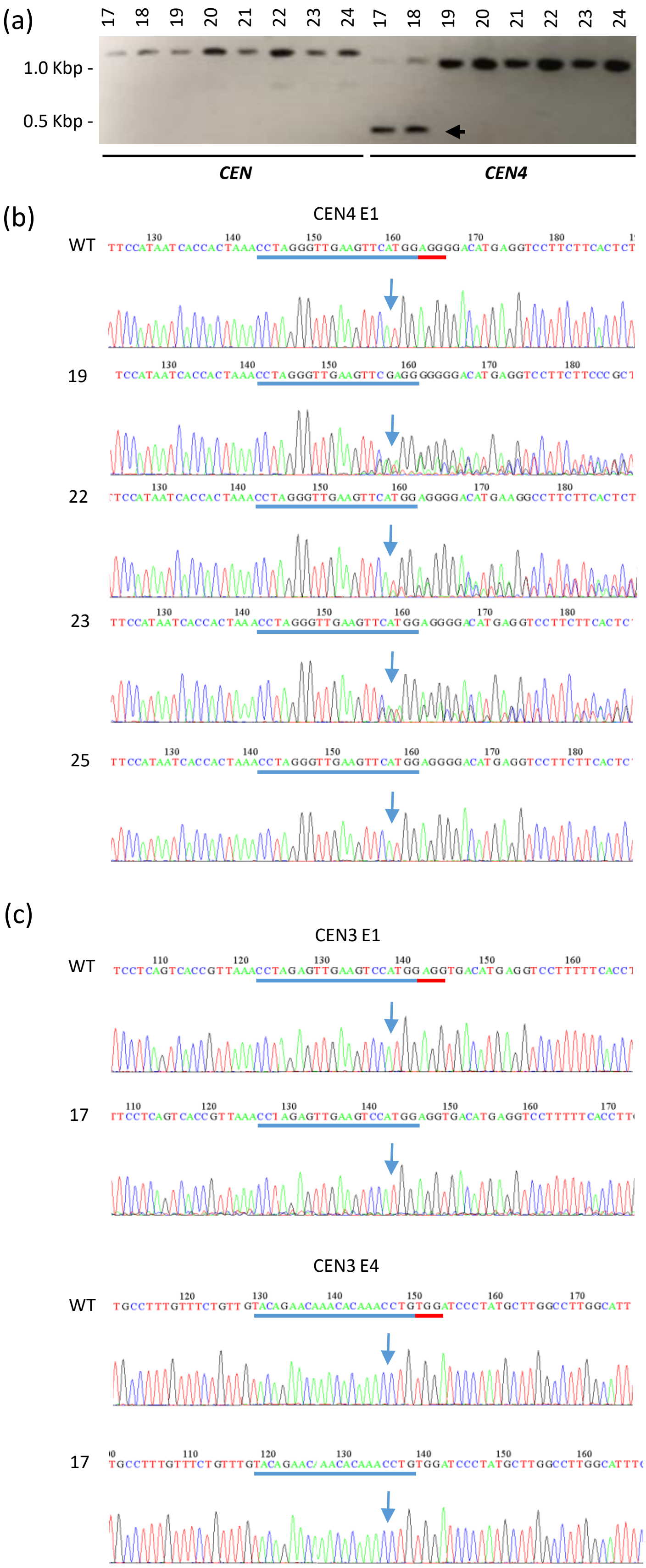

Supplementary Figure S2. Evaluation of CRISPR/Cas9-mediated gene editing in transgenic kiwifruit. (a) Example of amplification using gene-specific primers, which identified the E1-E4 deletion in *AcCEN4* in lines 17 and 18 (arrow). (b) Examples of wild-type (top) and edited *AcCEN4* E1 regions identified by sequencing of the PCR fragment. (c) Wild-type *AcCEN3* E1 and E4 regions identified by sequencing of the PCR fragment in early flowering line 17.

Figure S3

(a)

Line	*AcCEN4*	E1 … E4	*AcCEN*	E1 … E4
	WT	...CCTAGGGTTGAAGTTCATGG........CAGGTTTGTGTTTGTGCTCT...	WT	...CCAAGGGTTGAAGTTCATGG........CAGGTTTGTGTTTGTGCTCT...
U6-CEN4#14 *	14.DEL	...CCTAGGGTTGAAGTTCA--------------GTTTGTGTTTGTGCTCT...		
	CEN4.1	...CCTAGGGTTGAAGTTCAATGG.......CAG-----TGTTTGTGCTCT...	CEN.1	...CCAAGGGTTGAAGTTCATGG........CAG(156nt)GTTTGTGTTTGTGCTCT...
	CEN4.2	...CCTAGGGTTGAAGTTCAATGG.......CAG-----TGTTTGTGCTCT...	CEN.2	...CCAAGGGTTGAAGTTCATGG........CAG(156nt)GTTTGTGTTTGTGCTCT...
	CEN4.3	...CCTAGGGTTGAAGTTCAATGG.......CAG-TTT---------------.	CEN.3	...CCAAGGGTTGAAGTTCATGG........CAG(156nt)GTTTGTGTTTGTGCTCT...
	CEN4.4	...CCTAGGGTTGAAG---ATGG........CAG-TTT---------------.	CEN.4	...CCAAGGGTTGAAGTTCATGG........CAG(156nt)GTTTGTGTTTGTGCTCT...
U6-CEN4#18 **	18.DEL	...CCTAGGGTTGAAGTTCA--------------GTTTGTGTTTGTGCTCT...		
	CEN4.1	...CCTAGGGTTGAAG---ATGG........CAG-TTT---------------.	CEN.1	...CCAAGGGTTGAAGTT-ATGG........CAGAGTTTGTGTTTGTGCTCT...
	CEN4.2	...CCTAGGGTTGAAGTTCAATGG.......CAG-----TGTTTGTGCTCT...	CEN.2	...CCAAGGGTTGAAGTTCATGG........CAG----GTGTTTGTGCTCT...
	CEN4.3	...CCTAGGGTTGAAG---ATGG........CAG-TTT---------------.	CEN.3	...CCAAGGGTTGAAGTTCATGG........CAG----GTGTTTGTGCTCT...
	CEN4.4	...CCTAGGGTTGAAG---ATGG........CAG-TTT---------------.	CEN.4	...CCAAGGGTTGAAGTT-ATGG........CAGAGTTTGTGTTTGTGCTCT...
U6-CEN4#17 **	17.DEL	...CCTAGGGTTGAAGTTCA--------------GTTTGTGTTTGTGCTCT...		
	CEN4.1	...CCTAGGGTTGAAGTTCAATGG.......CAGGGTTTGTGTTTGTGCTCT...	CEN.1	...CCAAGGGTTGAAGTT-ATGG........CAGGTTTGTGTTTGTGCTCT...
	CEN4.2	...CCTAGGGTTGAAGTTCAATGG.......CAGGGTTTGTGTTTGTGCTCT...	CEN.2	...CCAAGGGTTGAAGTT-ATGG........CAGGTTTGTGTTTGTGCTCT...
	CEN4.3	...CCTAGGGTTGAAGTTCAATGG.......CAGGGTTTGTGTTTGTGCTCT...	CEN.3	...CCAAGGGTTGAAGTTCATGG........CAGGTTTGTGTTTGTGCTCT...
	CEN4.4	...CCTAGGGTTGAAGTTCAATGG.......CAGGGTTTGTGTTTGTGCTCT...	CEN.4	...CCAAGGGTTGAAGTTCATGG........CAGGTTTGTGTTTGTGCTCT...
U6-CEN4#7 ***	CEN4.1	...CCTAGGGTTGAAGTTCAATGG.......CAGGTTTGTGTTTGTGCTCT...	CEN.1	...CCAAGGGTTGAAGTTCATGG........CAGAGTTTGTGTTTGTGCTCT...
	CEN4.2	...CCTAGGGTTGAAGTTCAATGG.......CAGGTTTGTGTTTGTGCTCT...	CEN.2	...CCAAGGGTTGAAGTTCATGG........CAGAGTTTGTGTTTGTGCTCT...
	CEN4.3	...CCTAGGGTTGAAGTTCAATGG.......CAGGTTTGTGTTTGTGCTCT...	CEN.3	...CCAAGGGTTGAAGTTCATGG........CAGGTTTGTGTTTGTGCTCT...
	CEN4.4	...CCTAGGGTTGAAGTTCAATGG.......CAGGTTTGTGTTTGTGCTCT...	CEN.4	...CCAAGGGTTGAAGTTCATGG........CAGGTTTGTGTTTGTGCTCT...
U6-CEN4#22 ****	CEN4.1	...CCTAGGGTTGAAGTTCATGG........CAGGTTTGTGTTTGTGCTCT...	CEN.1	...CCAAGGGTTGAAGTTCATGG........CAGGTTTGTGTTTGTGCTCT...
	CEN4.2	...CCTAGGGTTGAAGTTCATGG........CAGGTTTGTGTTTGTGCTCT...	CEN.2	...CCAAGGGTTGAAGTTCATGG........CAGGTTTGTGTTTGTGCTCT...
	CEN4.3	...CCTAGGGTTGAAGTTCATGG........CAGGTTTGTGTTTGTGCTCT...	CEN.3	...CCAAGGGTTGAAGTTCATGG........CAGGTTTGTGTTTGTGCTCT...
	CEN4.4	...CCTAGGGT--------ATGG........CAGGTTTGTGTTTGTGCTCT...	CEN.4	...CCAAGGGTTGAAGTTCATGG........CAGGTTTGTGTTTGTGCTCT...
U6-CEN4#19 ****	CEN4.1	...CCTAGGGTTGAAGTTCATGG........CAGGTTTGTGTTTGTGCTCT...	CEN.1	...CCAAGGGTTGAAGTTCATGG........CAG---TGTGTTTGTGCTCT...
	CEN4.2	...CCTAGGGTTGAAGTT---GG........CAGGGTTTGTGTTTGTGCTCT...	CEN.2	...CCAAGGGTTGAAGTTCAATGG.......CAGGTTTGTGTTTGAGCTCT...
	CEN4.3	...CCTAGGGTTGAAGTTCATGG........CAGGTTTGTGTTTGTGCTCT...	CEN.3	...CCAAGGGTTGAAG-----GG........CAG-----TGTTTGTGCTCT...
	CEN4.4	...CCTAGGGTTGAAGTTCATGG........CAGGGTTTGTGTTTGTGCTCT...	CEN.4	...CCAAGGGTTGAAGTTCATGG........CAGGTTTGTGTTTGTGCTCT...

(b)

PTG-CEN4 ***

	g1	g2	g3	g4
WT *AcCEN4*	...CTAGTAGTTGGAAGAGTGAT.........	CATAATCACCACTAAACCTA.........	CCTTTGGTATGTATTAATTA.........	CTTCAGTACCCGGAAATTTG...
CEN4.1	...CTAGGTAGTTGGAAGAGTGAT........	CATAATCACCACTAAACCCTA........	CCT-------------ATTA.........	CTTCAGTACCCGGAAATTTG...
CEN4.2	...CTAGGTAGTTGGAAGAGTGAT........	CATAATCACCACTAAA-CTA.........	CCT-------------ATTA.........	CTTCAGTACCCGGAA-TTTG...
CEN4.3	...CTAGGTAGTTGGAAGAGTGAT........	CATAATCACCACTAAA-CTA.........	CCT-------------ATTA.........	CTTCAGTACCCGGAA-TTTG...
CEN4.4	...CTAGGTAGTTGGAAGAGTGAT........	CATAATCACCACTAAA-CTA.........	CCT-------------ATTA.........	CTTCAGTACCCGGAA-TTTG...
WT *AcCEN*	...TCCATAGTCACCACTAAACC.........	TCCTAGTGATCCATATCTGA.........	TCCTGTTGCTGCTGTGTTCT.........	TGTGTTCTTCAATGCCCAAA...
CEN.1	...TCCATAGTCACCACTAAACC.........	TCCTAGTGATCCATATCTGA.........	TCCTGTTGCTGCTGTGTTCT.........	TGTGTTCTTCAATGCCCAAA...
CEN.2	...TCCATAGTCACCACTAAACC.........	TCCTAGTGATCCATATCTGA.........	TCCTGTTGCTGCTGTGTTCT.........	TGTGTTCTTCAATGCCCAAA...
CEN.3	...TCCATAGTCACCACTAAACC.........	TCCTAGTGATCCATATCTGA.........	TCCTGTTGCTGCTGTGTTCT.........	TGTGTTCTTCAATGCCCAAA...
CEN.4	...TCCATAGTCACCACTAAACC.........	TCCTAGTGATCCATATCTGA.........	TCCTGTTGCTGCTGTGTTCT.........	TGTGTTCTTCAATGCCCAAA...

(c)

PTG-CEN ***

	g1	g2	g3	g4
WT *AcCEN4*	...CTAGTAGTTGGAAGAGTGAT.........	CATAATCACCACTAAACCTA.........	CCTTTGGTATGTATTAATTA.........	CTTCAGTACCCGGAAATTTG...
CEN4.1	...CTAGTAGTTGGAAGAGTGAT.........	CATAATCACCACTAAACCTA.........	CCTTTGGTATGTATTAATTA.........	CTTCAGTACCCGGAAATTTG...
CEN4.2	...CTAGTAGTTGGAAGAGTGAT.........	CATAATCACCACTAAACCTA.........	CCTTTGGTATGTATTAATTA.........	CTTCAGTACCCGGAAATTTG...
CEN4.3	...CTAGTAGTTGGAAGAGTGAT.........	CATAATCACCACTAAACCTA.........	CCTTTGGTATGTATTAATTA.........	CTTCAGTACCCGGAAATTTG...
CEN4.4	...CTAGTAGTTGGAAGAGTGAT.........	CATAATCACCACTAAACCTA.........	CCTTTGGTATGTATTAATTA.........	CTTCAGTACCCGGAAATTTG...
WT *AcCEN*	...TCCATAGTCACCACTAAACC.........	TCCTAGTGATCCATATCTGA.........	TCCTGTTGCTGCTGTGTTCT.........	TGTGTTCTTCAATGCCCAAA...
CEN.1	...TCCATAGTCACCACTAAACC.........	TCCTAGTGATC------TGA.........	TCC--TTGCTGCTGTGTTCT.........	TGTGTTCTTCAATGC--AAA...
CEN.2	...TCCA--GTCACCACTAAACC.........	TCCTAGTGATCCATA--TGA.........	TCCTTGTTGCTGCTGTGTTCT........	TGTGTTCTTCAATGCCC-AA...
CEN.3	...TCCATAGTCACCACTAAACC.........	TCCTAGTGATC------TGA.........	TCC--TTGCTGCTGTGTTCT.........	TGTGTTCTTCAATGC--AAA...
CEN.4	...TCCATAGTCACCACTAAACC.........	TCCTAGTGATC------TGA.........	TCC--TTGCTGCTGTGTTCT.........	TGTGTTCTTCAATGC--AAA...
CEN.5	...TCCATAGTCACCACTAAACC.........	TCCTAGTGATC------TGA.........	TCC--TTGCTGCTGTGTTCT.........	TGTGTTCTTCAATGC--AAA...

Supplementary Figure S3. Mutations (red font) identified in *AcCEN4* and *AcCEN* alleles in U6-CEN4 (a), PTG-CEN4 (b) and PTG-CEN (c) lines. At least four clones of gene-specific amplification products were subjected to sequence analysis. wt, wild-type; +, insertion; -, deletion.
